# Subtyping Somatic Tinnitus: A Cross-Sectional UK Cohort Study of Demographic, Clinical and Audiological Characteristics

**DOI:** 10.1371/journal.pone.0126254

**Published:** 2015-05-21

**Authors:** Jamie Ward, Claire Vella, Derek J. Hoare, Deborah A. Hall

**Affiliations:** 1 School of Psychology, University of Sussex, Brighton, BNI 9RH, United Kingdom; 2 National Institute for Health Research (NIHR) Nottingham Hearing Biomedical Research Unit, Nottingham, NG1 5DU, United Kingdom; 3 Otology and Hearing group, Division of Clinical Neuroscience, School of Medicine, University of Nottingham, Nottingham, NG7 2UH, United Kingdom; University of Texas at Dallas, UNITED STATES

## Abstract

Somatic tinnitus is the ability to modulate the psychoacoustic features of tinnitus by somatic manoeuvres. The condition is still not fully understood and further identification of this subtype is essential, particularly for the purpose of establishing protocols for both its diagnosis and treatment. This study aimed to investigate the characteristics of somatic tinnitus within a large UK cohort using a largely unselected sample. We believe this to be relatively unique in comparison to current literature on the topic. This was investigated by using a total of 608 participant assessments from a set of recognised tinnitus and audiology measures. Results from a set of chi-square tests of association found that amongst the individuals with somatic tinnitus, a higher proportion had pulsatile tinnitus (different from heartbeat), were under the age of 40, reported variation in the loudness of their tinnitus and reported temporomandibular joint (TMJ) disorder. The same pattern of results was confirmed using a multivariate analysis of the data based on logistic regression. These findings have strong implications towards the profiling of somatic tinnitus as a distinct subtype of general tinnitus.

## Introduction

Tinnitus is a common, and sometimes very distressing, long-term health condition that involves the sensation of a sound without any explanation of an external auditory stimulus. Idiopathic subjective tinnitus is where the experience of the phantom sound is heard by the individual alone and there is no known etiology. This is by far the most common form of tinnitus representing 95–98% of all tinnitus presentations. The most common risk factor for developing a subjective tinnitus is an otological problem leading to hearing loss, but ototoxic medication, head injury, orofacial, rheumatological, endocrine and metabolic problems may also play a causal role [1]. Objective tinnitus is the less common variant whereby an observer can hear the tinnitus. For objective tinnitus, often a medical diagnosis can be made which determines an internal sound source, such as pulsatile tinnitus caused by vascular origin, or myoclonus of the middle ear [2] or palatal muscles [3].

Given that otological conditions, especially hearing loss, present one of the major risk factors for tinnitus, neuroplastic responses to the reduction in sound input are deemed to play a pivotal role in the condition [4]. Sensory deafferentation affects the balance of excitatory and inhibitory activity leading to changes in the spontaneous stochastic neural firing rate, changes in the temporal firing pattern of otherwise stochastic spontaneous activity and spatiotopic reorganisation of frequency tuning (e.g. [5,6]). Changes are observed throughout the auditory nuclei within the ascending pathway, although it is still unresolved precisely what neural phenomenon is the correlate of the tinnitus sensation.

Synaptic plasticity related to tinnitus may not be limited to the central auditory system. Across the past several decades, clinical reports have described patients who are able to alter their tinnitus through somatic manoeuvres [7–9]. For instance, eye movements, jaw protrusion, jaw clenching, head and neck muscle contraction manoeuvres, or touch to the face or hands may modulate the loudness (or less commonly the pitch) of the tinnitus [10–13]. Several authors have put forward a physiological explanation for the somatosensory modulation of tinnitus—the dorsal cochlear nucleus (DCN) disinhibition model [8]. The fusiform cells of the DCN project directly to the central nucleus of the inferior colliculus as part of the extralemniscal auditory pathway [14]. These cells are unique in that they serve as a convergence point for integration of auditory input via cochlear nerve fibers with somatosensory input via the axons of cochlear nucleus granule cells [15–17]. These multisensory neurons are predisposed to cross-modal compensation following hearing loss. Findings within the animal literature indicate that projections from the trigeminal system to the DCN are increased and/or redistributed after hearing loss and can modulate spontaneous activity. Moreover, following noise-induced hearing loss, animals showed significantly lower thresholds, shorter latencies and durations, and increased amplitudes of response to trigeminal stimulation than their normal hearing counterparts. The finding that only neurons activated by trigeminal stimulation showed increased spontaneous rates after cochlear damage is indicative that somatosensory neurons may play a role in the pathogenesis of some forms of human tinnitus. The concept of disinhibition between sensory pathways is not restricted to tinnitus. It has also been influential in explaining other illusory percepts such as synaesthesia (e.g. experiencing vision from sound) and visual modulation of pain/touch phantom limb. In the case of phantom limb [18] and acquired synaesthesia involving blindness [19], the illusory experiences are invariably linked to sensory deafferentation, as seems to be the case for tinnitus.

In studying tinnitus in humans, the heterogeneity in terms of tinnitus aetiology, pathophysiology, and clinical features is well known. It is probable that different subtypes exist beyond the current subjective/objective distinction [20]. Indeed Baguley and colleagues [21] reiterated the challenge for an effective classification system informed by the underlying pathophysiological mechanisms. This would be a ground-breaking step towards personalised effective rehabilitation for individual tinnitus symptoms. Based on the finding that many patients can manipulate their tinnitus through somatic manoeuvres and the identification of neuronal pathways mediating somatosensory input to the DCN, the concept of ‘somatic tinnitus’ (or ‘somatosensory tinnitus’) has been proposed [7]. Whether somatic tinnitus is a clinically useful construct is not established. Nevertheless, Levine and colleagues [22] have proposed that treatment modalities directed toward the somatosensory system should be carefully assessed by targeting this tinnitus subgroup.

Perhaps with the exception of tinnitus in its objective form, one of the challenges to the international community is the lack of tools to effectively determine idiopathic subjective tinnitus subtyping. Individual studies invariably recruit only small numbers of patients. The lack of standardisation of core measures in the clinical history taking and evaluation of the condition makes it difficult to pool together data acquired by different studies to support meta-analysis of large datasets. The value of retrospective analysis of large datasets is illustrated in a recent exploratory study in which 1204 anonymised records were examined to determine whether comorbid orofacial complaints (specifically temporomandibular joint, TMJ, disorder) could provide a criterion for subtyping a somatic tinnitus [23]. Data presented in the form of descriptive statistics indicated that about 22% reported TMJ disorder. More people reporting TMJ problems could modulate their tinnitus by somatic manoeuvres than those without any TMJ problems (48% versus 30%, respectively). While these findings are interesting, they are somewhat preliminary as they report only the results of univariate descriptions of association and these are sensitive to confounding effects. Multivariate analyses would be needed to determine uniquely related variables because they take into account the interdependencies between different variables [24]. Another limitation relevant to the above review of the literature was that the data could not confirm a difference in hearing ability between tinnitus patients with and without TMJ complaints, perhaps because audiometric data were unavailable for about one-third of the cohort in the previous study [25]. Nevertheless, the authors made a case for conducting further studies in order to confirm these preliminary findings and to explore the relevance of the underlying TMJ pathology as a key defining variable for the putative somatic tinnitus subtype.

The objective of the current study is to investigate the characteristics of somatic tinnitus within a UK cohort. Unlike previous studies which have recruited from the clinical population (e.g. [23,24,25]), our cohort represents tinnitus in the general population. We first aim to describe the prevalence of somatic tinnitus in people without an objective tinnitus and second to determine whether these symptoms are linked to a particular profile of hearing loss, etiology, or other symptom characteristics using methods of model prediction. Our dataset has full audiometric profiles. These findings have implications towards the profiling of somatic tinnitus as a distinct subtype of tinnitus.

## Materials and Methods

### Participants

The meta-analysis used anonymised data that had been collected as part of various research studies led by the National Institute for Health Research (NIHR) Nottingham Hearing Biomedical Research Unit (BRU) conducted between 2009 and 2014. Because of the meta-analytic nature of this study it was not subject to a specific ethical approval, but for the individual research studies themselves written informed consent had been given by all participants and the studies had been approved by the following local National Health Service (NHS) Ethics Committees: Derbyshire [26]; East Midlands—Nottingham 1 [27,28]; East Midlands—Nottingham 2 [29], and East Midlands—Derby (Mohamad, personal communication). Sponsorship for all research studies was provided by Nottingham University Hospitals (NHS) Trust.

The majority of participants had been recruited in response to publicity in the national media and via the BRU website. A small proportion was recruited via poster advertisements in the Ear, Nose and Throat (ENT) and Audiology services at the Queen’s Medical Centre and Ropewalk House sites, Nottingham. All participants were assessed prior to any intervention as part of the eligibility screening or the baseline evaluation and so our dataset included data from people who had been excluded from the research study according to its specific eligibility criteria, as well as those who had been enrolled as a subject. The participants were: 82 participants from a randomised controlled study of auditory training using a computerised listening assessment platform [26]; 294 participants from a randomised controlled trial of a commercial tinnitus device [27]; 64 participants from a controlled study of auditory training using a computerised gameplay platform [29]; 60 participants from a prospective evaluation of hearing aid benefit for tinnitus with brain imaging as one of the secondary outcome measures [28], and 174 participants from an cross-sectional study evaluating the relationship between tinnitus and cognition (ongoing). This gave us a total of 674 participant records.

### Standardised data collection and data coding

In order to ensure comparability of data collected across different studies, the BRU uses a minimum of standardised assessments during assessment and evaluation of treatment efficacy (if relevant to the study). These core assessments consist of i) a detailed tinnitus and medical history reporting, ii) a global tinnitus severity estimate, and iii) audiometric thresholds at 0.5, 1.0, 2.0, and 4 kHz in the left and right ears.

History taking always followed the Tinnitus Sample Case History Questionnaire (TSCHQ) developed by international consensus [30]. The TSCHQ includes one question on somatic manoeuvres: specifically “Does any head and neck movement (e.g. moving the jaw forward or clenching the teeth), or having your arms/hands or head touched, affect your tinnitus?”. The TSCHQ allows a yes/no response, but we also added a ‘don’t know’ response option since these phenomena tend not to be known simply through self-introspection without a qualified clinician to perform the somatic manoeuvres during the assessment (see [24]). For the purpose of investigating somatic tinnitus, we focused on a selection of the most relevant TSCHQ items for analysis. In addition to a question about somatic manoeuvres known to alter the tinnitus, these included demographic characteristics (age, gender, handedness), tinnitus characteristics (duration, etiology, pulsatility, constancy, loudness) and comorbidities (hearing difficulty, vertigo, TMJ complaints). The exact wording of the questions used can be found in Table 1.

**Table 1:** Descriptive statistics for the sample of participants that was included in the univariate and multivariate analyses.

		n	%	*M*	*SD*
Gender (n = 608)	Male	411	67.6		
	Female	197	32.4		
Age (n = 608)	Under 40	67	11.0		
	40–49	106	17.4		
	50–59	159	26.2		
	60–69	193	31.7		
	70+	83	13.6		
	*Mean*			56.3	12.71
Handedness (n = 607)	Right	543	89.3		
	Left	55	9		
	Both	9	1.5		
Initial onset: When did you first experience your tinnitus? (n = 603)	0–5 years ago	226	37.5		
	6–20	257	42.6		
	21+	118	19.6		
	*Mean*			13.4	13.5
Was the initial onset of your tinnitus related to: (n = 607)	Change in hearing	45	7.4		
	loud sound	198	32.6		
	Whiplash or head trauma	23	3.8		
	Stress	39	6.4		
	Other	127	20.9		
	Don’t know	175	28.8		
Does your tinnitus seem to pulsate? (n = 608)	Yes (not heartbeat)	31	5.1		
	No	577	94.9		
How does your tinnitus manifest itself over time? (n = 606)	Intermittent	43	7.1		
	Constant	563	92.6		
Does the loudness of the tinnitus vary from day to day? (n = 608)	Yes	329	54.1		
	No	279	45.9		
Do you think you have a hearing problem? (n = 608)	Yes	407	66.9		
	No	201	33.1		
Do you suffer from vertigo or dizziness? (n = 608)	Yes	132	21.7		
	No	476	78.3		
Do you suffer from temporomandibular disorder? (n = 607)	Yes	49	8.1		
	No	558	91.9		
Global Tinnitus Severity; THI or THQ (n = 600)	Slight	114	19.0		
	Mild	228	38.0		
	moderate	147	24.5		
	Severe to catastrophic	111	18.5		
	*Mean (THI)*			34.7	21.1
	*Mean (THQ)*			38.9	17.2
Hearing Loss (Pure tone audiometry, PTA, n = 600)	None	335	55.8		
	Mild	206	34.3		
	Moderate to Severe	59	9.8		
	*Mean*			23.5	15.8

Statistics show demographic characteristics, tinnitus characteristics, reported comorbidities and assessment of hearing loss. Where ‘n’ does not sum to 608 this indicates missing data.

Global tinnitus severity was measured either using the Tinnitus Handicap Inventory (THI) [31] or the Tinnitus Handicap Questionnaire (THQ) [32], depending on the study. Both instruments yield a scaled score from 0 to 100. A decision was made to pool the scores to create a single continuous variable, given that these two outcome instruments show high convergent validity (r = 0.76) [33], have a similar (normal) distribution of scores and an almost identical clinically meaningful change score (20 and ≥21 respectively) [34,35].

Audiometric thresholds were converted into a pure tone average (PTA) at the four octave frequencies and this was then averaged across both ears to create a single continuous variable for the degree of hearing loss.

### Data coding and quality assessment

Three participant records were removed because the TSCHQ had not been administered. These were two from the study using a computerised gameplay platform [29] and one from the hearing aid study [28].

There were a small number of data re-codings for the TSCHQ. Five participants had given multiple answers in response to the question about etiology, and so for the purpose of analysis, a single etiology was coded. Events related to hearing loss (change in hearing or noise exposure) were maintained first, followed then by head or neck trauma events, stress related events and then lastly, other or unknown related events. In response to the question about constancy, 2 participants had selected both intermittent and constant and so these were coded as missing data because we could not be certain about the temporal attributes of the percept. Fifteen participants had given additional verbal descriptions in response to the question about TMJ disorder. Although they had not received a formal medical diagnosis, some did report experiencing some problems consistent with this. For 7 participants, we were therefore able to recode a TMJ disorder when a ‘no’ response was qualified by additional information about continuous jaw pain.

Following resolution of all data queries and data coding, we quantified the overall completeness of the dataset. In general, this was high with a total 0.53% of data missing. The lowest response rate was for the THI-THQ variable (1.94% missing).

## Results

Overall, 108 out of the 671 participants stated that they could manipulate their tinnitus through somatic manoeuvres. Thus the prevalence of the putative somatic tinnitus subtype in our research population of people with tinnitus was 16.1%.

### Participant characteristics

In order to characterise the putative subtype more precisely, two steps were taken to reduce unwanted heterogeneity across the cohort prior to any statistical analysis. First, records were removed if the participant did not know whether somatic movements affected the tinnitus (n = 14) or again did not complete this item (n = 2). Second, to eliminate participants who might have a diagnosis of objective (pulsatile) tinnitus of a neurovascular origin, we removed records for all those who confirmed that their tinnitus pulsated in rhythm with the heartbeat (n = 39) or did not complete this item (n = 8). In summary, 608 records remained. The demographics and descriptive statistics are summarised in Table 1 and the raw data can be found in S1 File.

Table 2 shows the prevalence of somatic tinnitus according to demographics (age, gender, handedness), tinnitus characteristics (duration, etiology, pulsatility, constancy, loudness), comorbidities (hearing difficulty, vertigo, TMJ complaints), global tinnitus severity and hearing loss classification from the PTA. For global tinnitus severity, the typical classifications for the THI were used (0–16 No handicap; 18–36 Mild; 38–56 Moderate; 58–100 Severe/catastrophic; [36]), combining severe and catastrophic into a single category and extending these categories to the THQ. For etiology, the separate options of ‘whiplash’ and ‘head injury’ were combined due to small numbers of responses in each category.

**Table 2:** Univariate analyses showing degree of association between reports of somatic modulation of tinnitus and other variables.

	% Somatic Modulation	χ^2^	*df*	*P*	*Cramer’s V*
**Age** (*under 40/40-49/50-59/60-69/70+*)	**26.9 / 15.1 / 18.2 / 13.5 / 9.6**	**9.98**	**4**	0.04*	**0.128**
Gender (*male/female*)	17.5 / 12.7	2.32	1	0.13	0.062
Handedness (*right/left/both*)	16.2 / 14.5 / 11.1	2.64	2	0.88	0.021
Tinnitus duration (*0-5/6-20/21+ years*)	16.8 / 17.0/ 11.9	1.81	2	0.41	.055
Etiology (*hearing change/ loud sound/ trauma/ stress / other/ don’t know*)	11.1 / 18.2 / 8.7 / 25.6 / 15.0 / 13.7	5.93	5	0.31	0.099
**Pulsatility** (*yes different from heartbeat/no*)	**35.5 / 14.9**	**9.29**	**1**	.002**	**0.124**
Constancy (*intermittent/constant*)	14 / 16	0.12	1	0.73	0.014
**Loudness** (*yes/no*)	**19.5 / 11.8**	**6.55**	**1**	0.01*	**0.104**
Hearing difficulty (*yes/no*)	14.5 / 18.9	1.95	1	0.16	0.057
Vertigo/dizziness (*yes/no*)	15.9 / 16**2**	0.00	1	0.99	0.001
**TMJ complaints** (*yes/no*)	**8.6 / 14.9**	**6.29**	**1**	0.01*	**0.102**
Global tinnitus severity (*slight/ mild/ moderate/ severe to catastrophic*)	9.6 / 17.9 / 15.0 / 18.9	4.94	3	0.18	0.091
PTA (*no HL/ mild HL/ moderate to severe HL*)	17.3/ 15.0 /6.8	4.30	2	0.12	0.085

Significant results are highlighted in bold, and effect sizes are given by Cramer’s V. Note

* p < .05

** p < .01

Model specification included univariate and multivariate analyses. Statistical analyses were conducted with IBM SPSS Statistics for Windows, Version 22.0.

### Univariate relationships with somatic modulation

The univariate approach was a set of chi-square tests of association performed between somatic modulation and the 13 independent variables listed in Table 2. For the univariate chi-square tests of association, age was converted into a categorical variable with five levels (<40, 40–49, 50–59, 60–69 and ≥70 years). Duration of tinnitus was converted into a categorical variable with three levels (0 to 5 years, 6–20 years, and 21 years or more). PTA was converted into a categorical variable with three levels (normal hearing defined as <25 decibel Hearing Level (dB HL), mild hearing loss defined as 25–44 dB HL, and moderate-severe hearing loss defined as ≥45 dB HL). The results are reported in Table 2.

Significant associations were found between somatic modulation and age, pulsatility, loudness and TMJ complaints. Individuals who could somatically modulate their tinnitus tended to be under the age of 40, have a pulsatile tinnitus (not synchronous with the heartbeat), reported variation in the tinnitus loudness from day to day and experienced TMJ complaints.

### Multivariate relationships with somatic modulation

Logistic regression analysis is advantageous because it takes into account the interdependencies between different variables and avoids the problem of multiple comparisons that is inherent with a series of independent chi-square tests of association. In addition, the variables of age, time since onset, tinnitus severity and PTA were treated as continuous data thus avoiding the data reduction that had been necessary in the univariate analysis.

For this method to be valid it is important to first check for multicollinearity between predictor variables. Collinearity statistics were run using presence of somatic modulation as dependent variable and the same 13 independent variables listed in Table 2. Collinearity problems are indicated by tolerance values below 0.1 or VIF (Variance Inflation Factor) values above 10 [37]. In the present dataset, the tolerance values ranged from 0.454 to 0.968 and the VIF values ranged from 1.033 to 2.204. As such, multicollinearity is not a concern and a logistic regression is appropriate.

A logistic regression analysis was performed using the same 13 independent variables considered previously. The stepwise (backwards) model using likelihood ratios is considered appropriate when the analysis is exploratory in nature [37]. This method initially includes all variables and then removes variables one by one (removing first the variable with the least impact on the model) until no further variables can be removed without having a substantial effect on how well the model fits the data. The final model removed eight variables and retained five. This represented a significant fit to the data (χ^2^ (5) = 26.02, *p* <. 001) and explained 7.5% (Nagelkerke’s *R* ^*2*^) of the variance. The results are summarised in Table 3. The model retained the variables of age, pulsatility, loudness variability, TMJ disorder and gender. The first four were identified in the univariate analyses. Gender did not come out as significant in the univariate analyses and was of borderline significance in the multivariate model (p =. 055).

**Table 3:** Logistic Regression Analysis of Somatically Modulated Tinnitus.

Independent Variable	B	SE	Wald	Sig.	Exp(B)
Age	.022	.009	5.959	.015	1.022
Gender	-.527	.275	3.668	.055	0.591
Pulsatility	-.983	.413	5.652	.017	0.374
Loudness varies	-.514	.246	4.367	.037	0.598
TMJ Disorder	-.858	.368	5.424	.029	0.424

Note that Exp(B) is typically used a measure of effect size in this analysis.

Finally, a confirmatory approach (based on the forced entry method) was taken specifying only the four independent variables that were significantly associated with somatic modulation in the chi-square tests of association (i.e. age, pulsatility, loudness variability, and TMJ complaints). The model was a significant fit to the data (χ ^2^ (4) = 24.40, *p* <. 001) and all four variables were significant predictors (age: Wald = 8.64, p =. 003; pulsatility: Wald = 4.39, p =. 036; loudness variability: Wald = 5.42, p =. 020; and TMJ disorder: Wald = 4.59, p =. 032).

## Discussion

The main findings of this study are twofold:

- The prevalence of self-reported ability to modulate tinnitus through somatic manoeuvres was 16% in a general cohort of people with tinnitus. We define this as somatic tinnitus.
- Somatic tinnitus was significantly predicted by age, pulsatility, loudness and TMJ complaints.

There is little agreement about the prevalence of somatic modulation of tinnitus and except for a handful of dedicated specialists it has been largely neglected by clinicians and researchers over the years. Estimates of self-reported somatic tinnitus in the present study are comparable to at least one previous report that we could find in the published literature. Notably, within a specialist USA tinnitus clinic, about 20% of patients reported at interview that they could somatically modulate their tinnitus by head and neck movements or by muscle contractions, such as clenching the teeth together [38]. Prevalence is higher in cohorts where the assessment has involved the individual performing a guided series of different forceful contractions and judging its effect on loudness, pitch or lateralisation. For example, Levine [8] found that 68% of 70 consecutive patients seen in the clinic could somatically modulate their tinnitus. A similar picture is seen in clinical samples from Brazil [12] and Korea and Vietnam [39]. An even higher prevalence of 80% was reported in a nonclinical sample of people with tinnitus [40]. Again however, the manner in which somatic modulation was ascertained was by forceful head and neck contractions manipulated by a specialist neurologist and his team. Hence, the prevalence of somatic tinnitus is highly dependent upon whether self-report or expert guided manoeuvres are conducted. Self-report is likely to under-diagnose the condition. Nevertheless, the current study provides a conservative estimate based on a widely used screening questionnaire [30].

The results suggest that somatic tinnitus may represent a distinct sub-type. Whereas tinnitus in general is linked to older age and hearing loss [1], our results suggest that somatic tinnitus is more common in the younger group, but is not related to the severity of hearing loss or the severity of tinnitus itself. Moreover, the results suggest comorbidity with TMJ disorder. Whereas our study contrasted people with tinnitus who reported somatic modulation and found an association with TMJ disorder, Vielsmeier et al. [23] took the complementary approach of contrasting tinnitus with and without TMJ disorder and noted an association with somatic modulation. Their tinnitus group with TMJ disorder were also likely to be younger and female. A female bias for somatic modulation was also reported by Won et al. [39] but was not found in the present study (a trend was found in the reverse direction). However, an even gender distribution for somatic modulation still stands in contrast to the strongly male bias for tinnitus in general as found in our sample (67.6%) and elsewhere [1]. Symptomatic temporomandibular joint disk displacement peaks in teenage years and rarely onsets in those aged >45 [41]. Somatic causes may be less apparent in the older sample where tinnitus is more readily associated with hearing loss which onsets in the natural course of aging progress from aged 50 onwards. The onset of TMJ disorder is also linked to stressful life events [42]. Although we didn’t find a statistically significant association with stress it was the largest numerical etiological predictor of somatic modulation.

The putative mechanism for somatic tinnitus is a changed profile of inhibitory or excitatory connections between the auditory and somatosensory pathways. In the model of Shore and colleagues [43], increased somatosensory input reflects disinhibition in order to compensate for hearing loss. This model predicts a positive relationship between hearing loss and degree of somatic modulation that we failed to observe. Instead, somatic modulation may be related to changes in sensitivity within the somatosensory system (either in addition to or instead of such changes within the auditory system) such as those which characterise TMJ disorder (e.g.[44,45]). Future research in this area should explore the somatosensory profile of people with tinnitus in addition to those relating to hearing. For instance, temporomandibular disorder has two broad sub-types: one characterised by symptoms of pain and one characterised by intra-articular symptoms such as clicking of the jaw [46]. Knowing which of these symptom constellations is linked to tinnitus is important both clinically and theoretically. For instance, the trigeminal pathway to the DCN carries information about touch and proprioception but not pain [43] so this theory predicts that pain is unlikely to be the driving symptom. However, there are other cortico-cortical pathways which are sensitive to pain (in addition to touch and proprioception) that could potentially give rise to somatic tinnitus. Patients with TMJ disorder activate, in fMRI, the primary auditory cortex in response to tactile stimuli and this has been linked to connections between this region and the adjacent secondary somatosensory cortex [47]. In healthy controls, there are anatomical connections between these brain regions and, in a single case study of somatosensory impairment following thalamic lesion, these pathways became both structurally and functionally altered giving rise to auditory-induced somatic sensations [48]. The latter is conceptually opposite to somatically modulated/ induced tinnitus.

In general our findings have significant implications for the subtyping of tinnitus. Subtyping of tinnitus is important diagnostically, and in terms of improving treatment, and enhancing our theoretical understanding. Our findings provide evidence for a profile of somatic tinnitus. Theoretical models of tinnitus need to consider not only the integrity of the auditory system but also the integrity of, and its inter-relation with, the other senses.

## Supporting Information

S1 File The raw data for the main analyses. This relates to 608 cases of tinnitus(in rows) with the answers to the different questions (in columns).
(ZIP)

## References

[1] Hoffman H, Reed G (2004) Epidemiology of tinnitus In: Snow J, editor. Tinnitus: Theory and Management London: BC Decker.

[2] Bento RF, Sanchez TG, Miniti A (1998) Continuous high frequency objective tinnitus caused by middle ear myoclonus. Ear Nose and Throat Journal 77: 814–818. 9818532

[3] Seidman MD, Arenberg JG, Shirwany NA (1999) Palatal myoclonus as a cause of objective tinnitus: a report of six cases and a review of the literature. Ear, nose, & throat journal 78: 292–294, 296–297. 10224704

[4] Eggermont J, Roberts L (2004) The neuroscience of tinnitus. Trends in Neurosciences 27: 676–682. 15474168 10.1016/j.tins.2004.08.010

[5] Adjamian P, Sereda M, Hall DA (2009) The mechanisms of tinnitus: Perspectives from human functional neuroimaging. Hearing Research 253: 15–31. 10.1016/j.heares.2009.04.001 19364527

[6] Roberts LE, Eggermont JJ, Caspary DM, Shore SE, Melcher JR, Kaltenbach JA (2010) Ringing ears: The neuroscience of tinnitus. Journal of Neuroscience 30: 14972–14979. 10.1523/JNEUROSCI.4028-10.2010 21068300 PMC3073522

[7] Levine RA (1999) Somatic (Craniocervical) tinnitus and the dorsal cochlear nucleus hypothesis. American Journal of Otolaryngology 20: 351–362. 10609479 10.1016/s0196-0709(99)90074-1

[8] Levine RA. Somatic modulation appears to be a fundamental attribute of tinnitus In: Hazell J, editor; 1999; Cambridge, UK The British Society of Audiology.

[9] Simmons R, Dambra C, Lobarinas E, Stocking C, Salvi R (2008) Head, Neck, and Eye Movements That Modulate Tinnitus. Seminars in Hearing 29: 361–370. 19183705 10.1055/s-0028-1095895 PMC2633109

[10] Coad ML, Lockwood A, Salvi R, Burkard R (2001) Characteristics of patients with gaze-evoked tinnitus. Otology & Neurotology 22: 650–654. 11568674 10.1097/00129492-200109000-00016

[11] Levine RA, Abel M, Cheng H (2003) CNS somatosensory-auditory interactions elicit or modulate tinnitus. Experimental Brain Research 153: 643–648. 14600798 10.1007/s00221-003-1747-3

[12] Sanchez TG, Lima AdS, Brandao AL, Lorenzi MC, Bento RF (2007) Somatic modulation of tinnitus: Test reliability and results after repetitive muscle contraction training. Annals of Otology Rhinology and Laryngology 116: 30–35. 17305275 10.1177/000348940711600106

[13] Sanchez TG, Rocha CB (2011) Tinnitus caused and influenced by the somatosensory system; Moller AR, Langguth B, DeRidder D, Kleinjung T, editors: Springer. 363–368 p.

[14] Møller AR, Møller MB, Yokota M (1992) Some forms of tinnitus may involve the extralemniscal auditory pathway. Laryngoscope 102: 1165–1171. 1405968 10.1288/00005537-199210000-00012

[15] Shore SE (2005) Multisensory integration in the dorsal cochlear nucleus: unit responses to acoustic and trigeminal ganglion stimulation. European Journal of Neuroscience 21: 3334–3348. 16026471 10.1111/j.1460-9568.2005.04142.x

[16] Shore SE, Koehler S, Oldakowski M, Hughes LF, Syed S (2008) Dorsal cochlear nucleus responses to somatosensory stimulation are enhanced after noise-induced hearing loss. European Journal of Neuroscience 27: 155–168. 10.1111/j.1460-9568.2007.05983.x 18184319 PMC2614620

[17] Shore SE, Vass Z, Wys NL, Altschuler RA (2000) Trigeminal ganglion innervates the auditory brainstem. Journal of Comparative Neurology 419: 271–285. 10723004 10.1002/(sici)1096-9861(20000410)419:3<271::aid-cne1>3.0.co;2-m

[18] Ramachandran VS, Hirstein W (1998) The perception of phantom limbs. Brain 121: 1603–1630. 9762952 10.1093/brain/121.9.1603

[19] Afra M, Funke M, Matsuo F (2009) Acquired auditory-visual synesthesia: A window to early cross-modal sensory interactions. Psychology Research and Behavior Management 2: 31–37. 22110319 10.2147/prbm.s4481 PMC3218766

[20] Landgrebe M, Zeman F, Koller M, Eberl Y, Mohr M, Reiter J, et al (2010) The Tinnitus Research Initiative (TRI) database: A new approach for delineation of tinnitus subtypes and generation of predictors for treatment outcome. Bmc Medical Informatics and Decision Making 10. 10.1186/1472-6947-10-42 PMC2920857 20682024

[21] Baguley D, McFerran D, Hall DA (2013) Tinnitus. The Lancet 382: 1600–1607. 10.1016/S0140-6736(13)60142-7 23827090

[22] Levine RA, Nam EC, Oron Y, Melcher JR (2007) Evidence for a tinnitus subgroup responsive to somatosensory based treatment modalities In: Langguth B, Hajak G, Kleinjung T, Cacace A, Moller AR, editors. Tinnitus: Pathophysiology and Treatment pp. 195–207. 10.1016/S0079-6123(07)66017-8 17956783

[23] Vielsmeier V, Strutz J, Kleinjung T, Schecklmann M, Kreuzer PM, Landgrebe M, et al (2012) hoeTemporomandibular joint disorder complaints in tinnitus: Further hints for a putative tinnitus subtype. Plos One 7. 10.1371/journal.pone.0038887 PMC3378537 22723902

[24] Hoekstra CEL, Wesdorp FM, van Zanten GA (2014) Socio-demographic, health, and tinnitus related variables affecting tinnitus severity. Ear and Hearing 35: 544–554. 10.1097/AUD.0000000000000045 25003528

[25] Vielsmeier V, Kleinjung T, Strutz J, Buergers R, Kreuzer PM, Langguth B (2011) Tinnitus with temporomandibular joint disorders: a specific entity of tinnitus patients?. Otolaryngology-Head and Neck Surgery 145: 748–752. 10.1177/0194599811413376 21705788

[26] Hoare DJ, Kowalkowski VL, Hall DA (2012) Effects of frequency discrimination training on tinnitus: results from two randomised controlled trials. Journal of the Association for Research in Otolaryngology 13: 543–559. 10.1007/s10162-012-0323-6 22476724 PMC3387303

[27] Hoare DJ, Pierzycki RH, Thomas H, McAlpine D, Hall DA (2013) Evaluation of the acoustic aoordinated reset(R) neuromodulation therapy for tinnitus: study protocol for a double-blind randomised placebo-controlled trial. Trials 14: 207 10.1186/1745-6215-14-207 23842505 PMC3710266

[28] Davies JJ, Gander PE, Andrews M, Hall DA (2014) Auditory network connectivity in tinnitus patients: a resting-state fMRI study. International Journal of Audiology 53: 192–198. 10.3109/14992027.2013.846482 24200464 PMC3956492

[29] Hoare DJ, Van Labeke N, McCormack A, Sereda M, Smith S, Al Taher H, et al (2014) Gameplay as a source of intrinsic motivation in a randomized controlled trial of auditory training for tinnitus. Plos One 9. 10.1371/journal.pone.0107430 PMC4162598 25215617

[30] Langguth B, Goodey R, Azevedo A, Bjorne A, Cacace A, Crocetti A, et al (2007) Consensus for tinnitus patient assessment and treatment outcome measurement: Tinnitus Research Initiative meeting, Regensburg, July 2006. Tinnitus: Pathophysiology and Treatment 166: 525–536. 17956816 10.1016/S0079-6123(07)66050-6 PMC4283806

[31] Newman CW, Jacobson GP, Spitzer JB (1996) Development of the tinnitus handicap inventory. Archives of Otolaryngology-Head & Neck Surgery 122: 143–148. 8630207 10.1001/archotol.1996.01890140029007

[32] Kuk FK, Tyler RS, Russell D, Jordan H (1990) The psychometric properties of a tinnitus handicap questionnaire. Ear and Hearing 11: 434–445. 2073977 10.1097/00003446-199012000-00005

[33] Robinson SK, McQuaid JR, Viirre ES, Betzig LL, Miller DL, Bailey KA, et al (2003) Relationship of tinnitus questionnaires to depressive symptoms, quality of well-being, and internal focus. The International Tinnitus Journal 9: 97–103. 15106282

[34] Newman CW, Wharton JA, Jacobson GP (1995) Retest stability of the tinnitus handicap questionnaire. The Annals of Otology, Rhinology and Laryngology 104: 718–723. 7661523 10.1177/000348949510400910

[35] Newman CW, Sandridge SA, Jacobson GP (1998) Psychometric adequacy of the Tinnitus Handicap Inventory (THI) for evaluating treatment outcome. Journal of the American Academy of Audiology 9: 153–160. 9564679

[36] McCombe A, Baguley D, Coles R, McKenna L, McKinney C, Windle-Taylor P (2001) Guidelines for the grading of tinnitus severity: the results of a working group commissioned by the British Association of Otolaryngologists, Head and Neck Surgeons, 1999. Clinical Otolaryngology 26: 388–393. 11678946 10.1046/j.1365-2273.2001.00490.x

[37] Field A (2005) Discovering Statistics using SPSS—Second Edition London: Sage.

[38] Levine RA, Kiang NYS (1995) A conversation about tinnitus In: Vernon J, Møller AR, editors. Mechanisms of tinnitus. Boston: Allyn and Bacon pp. 149–162.

[39] Won JY, Yoo S, Lee SK, Choi HK, Yakunina N, Quang L, et al (2013) Prevalence and factors associated with neck and jaw muscle modulation of tinnitus. Audiology and Neuro-Otology 18: 261–273. 10.1159/000351685 23881235

[40] Abel MD, Levine RA (2004) Muscle contractions and auditory perception in tinnitus patients and nonclinical subjects. Cranio-the Journal of Craniomandibular Practice 22: 181–191. 15293775 10.1179/crn.2004.024

[41] Isberg A, Hägglund M, Paesani D (1998) The effect of age and gender on the onset of symptomatic temporomandibular joint disk displacement. Oral Surgery, Oral Medicine, Oral Pathology, Oral Radiology, and Endodontology 85: 252–257. 9540079 10.1016/s1079-2104(98)90004-x

[42] Speculand B, Hughes AO, Goss AN (1984) Role of recent stressful life events experience in the onset of TMJ dysfunction pain. Community Dentistry and Oral Epidemiology 12: 197–202. 6589114 10.1111/j.1600-0528.1984.tb01439.x

[43] Shore S, Zhou J, Koehler S (2007) Neural mechanisms underlying somatic tinnitus In: Langguth B, Hajak G, Kleinjung T, Cacace A, Moller AR, editors. Tinnitus: Pathophysiology and Treatment pp. 107-+.

[44] Maixner W, Fillingim R, Sigurdsson A, Kincaid S, Silva S (1998) Sensitivity of patients with painful temporomandibular disorders to experimentally evoked pain: evidence for altered temporal summation of pain. Pain 76: 71–81. 9696460 10.1016/s0304-3959(98)00028-1

[45] Woolf CJ (2011) Central sensitization: Implications for the diagnosis and treatment of pain. Pain 152: S2–S15. 10.1016/j.pain.2010.09.030 20961685 PMC3268359

[46] Schiffman E, Ohrbach R, Truelove E, Look J, Anderson G, Goulet J-P, et al (2014) Diagnostic criteria for temporomandibular disorders (DC/TMD) for clinical and research applications: recommendations of the international RDC/TMD consortium network and orofacial pain special interest group. Journal of Oral & Facial Pain and Headache 28: 6–27. 24482784 10.11607/jop.1151 PMC4478082

[47] Nebel MB, Folger S, Tommerdahl M, Hollins M, McGlone F, Essick G (2010) Temporomandibular disorder modifies cortical response to tactile stimulation. Journal of Pain 11: 1083–1094. 10.1016/j.jpain.2010.02.021 20462805 PMC2943972

[48] Beauchamp MS, Ro T (2008) Neural substrates of sound-touch synesthesia after a thalamic lesion. Journal of Neuroscience 28: 13696–13702. 10.1523/JNEUROSCI.3872-08.2008 19074042 PMC6671766

